# Poor Performance in SNF-VBP Program is Associated with Worse COVID-19 Outcomes in Nursing Homes

**DOI:** 10.1093/geroni/igab046.3632

**Published:** 2021-12-17

**Authors:** Jennifer Gaudet Hefele, Matt Aldag, Riad Elmor, Charanya Kaushik, Jessica Simpson Ballard

**Affiliations:** 1 Booz Allen Hamilton, Hollis, New Hampshire, United States; 2 Booz Allen Hamilton, Bethesda, Maryland, United States

## Abstract

Skilled Nursing Facility Value-Based Purchasing (SNF-VBP) was a new Medicare payment program when COVID-19 began. SNF-VBP aims to improve care through payment bonuses and penalties. However, studies have shown that minority-serving nursing homes (NHs) tend to fare worse under SNF-VBP (more likely to receive penalties, less likely to receive bonuses). This study sought to examine whether SNF-VBP performance prior to the pandemic was associated with COVID-19 outcomes and whether associations varied in NHs where the majority of residents are Black/African American (majority-Black/AA). Using publicly available data on COVID-19 outcomes and vaccinations, SNF-VBP performance, and NH characteristics, we found that majority-Black/AA NHs were less likely to have zero infections; had higher case fatality rates; and had lower resident and staff vaccinations rates compared to NHs where the majority of residents are White. Across all NHs, worse SNF-VBP performance was associated with worse COVID-19 outcomes (the bottom quintile of SNF-VBP performers were more likely to experience COVID-19 infections and had lower vaccination rates; the highest performers had higher vaccination rates). However, in stratified analyses, SNF-VBP performance was not significantly associated with COVID-19 outcomes in majority-Black/AA NHs compared with majority-White NHs. The association between poor SNF-VBP performance and poor COVID-19 outcomes is concerning. Overall findings suggest that SNF-VBP performance prior to the pandemic is an important indicator of subsequent COVID-19 outcomes. However, it is unclear whether poor SNF-VBP performance is signaling overall poor quality or whether it is signaling a financial disadvantage caused by the program itself that in turn impacted COVID-19 outcomes.

